# Molecular Characterization and Assessment of Risk Factors Associated with *Theileria annulata* Infection

**DOI:** 10.3390/microorganisms10081614

**Published:** 2022-08-09

**Authors:** Karim Ullah, Muhammad Numan, Abdulaziz Alouffi, Mashal M. Almutairi, Hafsa Zahid, Majid Khan, Zia Ul Islam, Atif Kamil, Sher Zaman Safi, Haroon Ahmed, Tetsuya Tanaka, Abid Ali

**Affiliations:** 1 Department of Zoology, Abdul Wali Khan University Mardan, Mardan 23200, Pakistan; 2 King Abdulaziz City for Science and Technology, Riyadh 12354, Saudi Arabia; 3 Department of Pharmacology and Toxicology, College of Pharmacy, King Saud University, Riyadh 11451, Saudi Arabia; 4 Department of Biotechnology, Abdul Wali Khan University Mardan, Mardan 23200, Pakistan; 5 Faculty of Medicine, Bioscience and Nursing, MAHSA University, Jenjarom 42610, Selangor, Malaysia; 6 Department of Biosciences, COMSATS University Islamabad (CUI), Park Road, Chak Shahzad, Islamabad 45550, Pakistan; 7 Laboratory of Infectious Diseases, Joint Faculty of Veterinary Medicine, Kagoshima University, 1-21-24 Korimoto, Kagoshima 890-0065, Japan

**Keywords:** cattle, *Theileria annulata*, risk factors, *18S rRNA*, Pakistan

## Abstract

**Simple Summary:**

*Theileria* is the tick-borne disease-causing agent responsible for theileriosis in various animals. Like other tick-borne pathogens, the epidemiology, associated risk factors and genetic diversity of *Theileria* spp. have mostly remained unexplored in the region. The current study was designed to analyze the epidemiology, associated risk factors, and molecular characterization of *Theileria* spp. in the selected districts of Khyber Pakhtunkhwa, Pakistan. Among the chosen districts, the highest infection was found in Upper Dir, while the most prevalent species was *Theileria annulata* clustered with sequences for the same species reported from Pakistan, China, and Italy. The risk factors associated with *Theileria* infection included age, gender, breeds, feeding system, hygienic measure, farming system, stall system, and seasons. The microscopic examination, in combination with the molecular approach, will enhance the early diagnosis and accurate identification of *Theileria* spp. and facilitate effective control strategies against these parasites.

**Abstract:**

*Theileria annulata* is a tick-associated parasite that causes tropical theileriosis in livestock and is responsible for huge economic losses. Studies have been neglected on the effect of *Theileria* spp. on cattle in Khyber Pakhtunkhwa (KP), Pakistan. The present study was designed to determine the genetic diversity and assess the risk factors associated with *Theileria* infection in selected districts of KP. Information on the risk factors related to the *Theileria* infection was collected through a questionnaire. Blood samples were collected from symptomatic cattle from January 2019 to February 2020, identified morphologically through microscopic examination, and processed for molecular characterization using the *18S rRNA* gene as a genetic marker. Of the 555 cattle examined (136, 24.5%) and (294, 53%) were found positive for *Theileria* spp. by microscopic examination and a PCR test, respectively. Based on the PCR test, the highest prevalence of infection was found in district Upper Dir (46/75, 61.3%), followed by Lower Dir (54/90, 60%); Malakand (51/88, 57.9%); Peshawar (40/80, 50%); and Charsadda (52/112, 46.4%), with the lowest in Bajaur (51/110, 46.34%). A BLAST analysis of the *18S rDNA* sequences showed 99.5% identity with *T. annulata*. In a phylogenetic tree, the *18S rDNA* sequence of *T. annulata* clustered with sequences from Pakistan, China, and Italy. A significant association was observed between the prevalence of infection and different host characteristics. The highest infection was found in adult cattle (216/360, 60%); females (218/377, 57.8%); and Holstein Friesian (120/180, 66.6%). *Theileria* infection was significantly associated with management practices. Higher infection rates were observed in free-grazing cattle (190/412, 42.2%); those kept in unhygienic conditions (246/405, 60.7%); cattle in combined farming systems (165/255, 64.8%); and those in congested stall systems (150/218, 68.8%). Seasonal patterns were found to be significantly associated with infection, and a higher infection rate was observed in summer (215/350, 61.4%) than in winter (79/205, 38.5%). Identified risk factors should be considered in designing practical control approaches to reduce the burden of *Theileria* infection. Large scale studies are required to explore the diversity of *Theileria* species in KP, Pakistan.

## 1. Introduction

Tick-borne pathogens cause diseases and affect millions of domesticated and wild animals in tropical and subtropical regions of the world [1,2]. Theileriosis is one of the most common tick-borne infection, and approximately 250 million animals are at risk annually [2]. Theileriosis occurs worldwide, affects nearly all ungulates, and causes either latent or lethal infections with high morbidity and mortality [2,3]. Among the different species of *Theileria*, *Theileria annulata* and *Theileria parva* are the most pathogenic for cattle, while other *Theileria* species are comparatively less pathogenic; however, all these species are well-known as life-threatening risks to various vertebrates [4]. Several species of *Theileria* are endemic in Pakistan, including *T. annulata*, *Theileria orientalis*, *Theileria lestoquardi*, and *Theileria ovis* [5,6,7].

Due to the morphological resemblances among *Theileria* spp., high technical expertise is required to differentiate these species by microscopic examination [1,8,9,10]. Several approaches such as the conventional methods of microscopic examination [1]; xenodiagnosis [11]; serological assays such as blood indirect fluorescent antibody (IFAT) [12] and enzyme-linked immunosorbent assay (ELISA) [13], as well as molecular assays such as polymerase chain reaction (PCR) [1,14]; loop-mediated isothermal amplification (LAMP); reverse line blot (RLB) [15]; restriction fragment length polymorphism (RFLP) [16]; and DNA sequencing have been used for the accurate detection and differentiation of these pathogens. Among them, molecular approaches are well known for detecting and identifying various *Theileria* spp. and their genotypes [17]. The *18S rRNA* gene has been utilized effectively to detect and identify various *Theileria* spp. [1,18]. Moreover, this gene has been sequenced and used in the evolutionary analysis of many *Theileria* species from various regions [19]. The currently available techniques are restricted in specificity and sensitivity, and accurate positive detection may be performed by multiple assays [1].

In Pakistan, rapid population growth has forced the government to import different cross-breed cattle with a high capacity for milk and meat production, but these cattle are susceptible to *Theileria* infection [2,20]. The cross-breeding of exotic animals with local animals has increased the susceptibility of these animals to several life-threatening diseases, including theileriosis [5,19]. Moreover, favorable agro-ecological conditions for tick vectors (*Hyalomma* and *Rhipicephalus* species) in Pakistan, such as humidity, temperature, long summer, and rainfall, play a major role in the propagation of these infectious agents [2,19,21,22,23]. The surveillance of these infectious agents is crucial for the timely control of outbreaks. Keeping in view the economic impact and importance of livestock in the country, livestock contributed approximately 61.9% of agricultural value added and 14.0% to the national GDP during 2021–2022 [Pakistan Economic Survey 2021–2022], The current study was intended to estimate the prevalence of *Theileria* infection and assess the associated risk factors and molecular characterization of *Theileria* species infecting cattle in Khyber Pakhtunkhwa (KP), Pakistan.

## 2. Materials and Methods

### 2.1. Ethical Approval

The ethical approval for this study was obtained from the Advanced Studies Research Board (ASRB) members, AWKUM/2020/4871, Faculty of Chemical and Life Sciences, Abdul Wali Khan University Mardan, KP, Pakistan.

### 2.2. Sample Collection and Study Area

**Figure 1:**
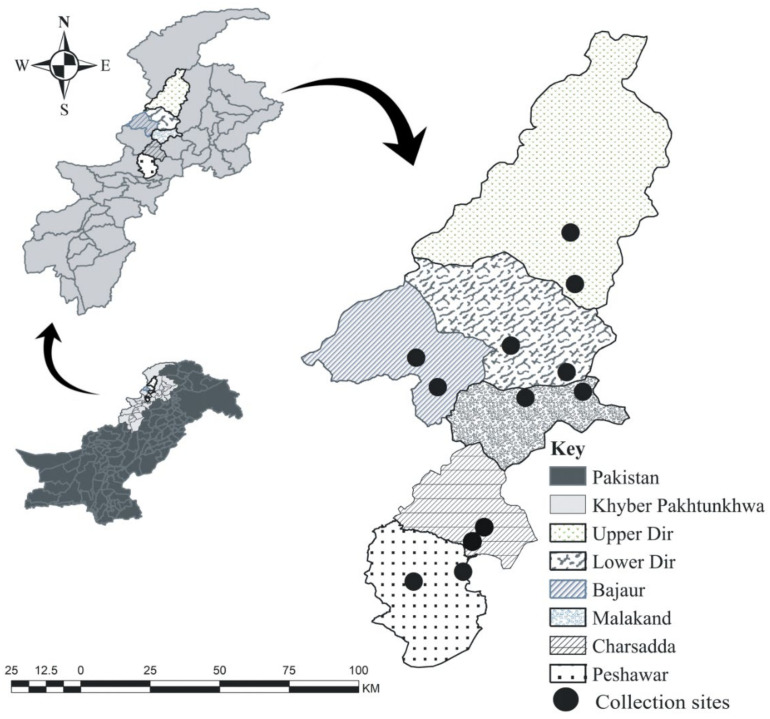
Map showing the sites where blood samples were collected in the selected districts of Khyber Pakhtunkhwa.

From January 2019 to February 2020 the blood samples were collected from the jugular vein of symptomatic cattle by a disposable 5 mL sterile syringe. The samples were randomly collected from various cattle herds in six districts, including: Peshawar (34°00′20.2″ N, 71°34′00.8″ E); Charsadda (34°10′02.0″ N, 71°45′21.2″ E); Malakand (34°33′07.4″ N, 71°53′37.1″ E); Lower Dir (34°54′05.8″ N, 71°48′51.2″ E); Upper Dir (35°19′00.1″ N, 72°03′57.2″ E); and Bajaur (34°45′43.2″ N, 71°32′18.0″ E) of KP, Pakistan. For further analyses, the collected blood samples were kept in 5.0 mL EDTA tubes at −30 °C. The geographic coordinates for the collection sites were obtained using “Google Earth pro”, and the map was designed by ArcGIS V. 10.3.1 (ESRI, Redlands, CA, USA) (Figure 1). While collecting the blood samples, relevant information such as age, gender, breeds, feeding system, hygienic measures, farming system, stall system, and collection seasons was collected using a questionnaire (Supplementary File S1).

### 2.3. Microscopic Examination and Clinical Signs

Blood samples collected from symptomatic cattle were examined microscopically using thin blood smears stained with Giemsa stain (8–10%) [24] and examined by using an oil immersion lens at 100× magnification using a microscope (BIOBASE, XS-208A, Jinan, China). The presence of any *Theileria* spp. was checked based on morphology [25]. The symptomatic cattle hosts were observed with clinical signs such as fever, loss of appetite, nasal and ocular discharge, oral lesions, diarrhea, anorexia, and reduced milk production.

### 2.4. DNA Extraction and PCR

Genomic DNA was extracted from each collected blood (750 μL) sample using the standard phenol-chloroform protocol, and the DNA pellet was hydrated by adding 50 μL of “nuclease-free” water [26,27,28]. The extracted genomic DNA samples were quantified via Nano-Q (Optizen, Daejeon, Korea) and stored at −20 °C for further analysis. A pair of species-specific primers (forward: 5′-GGCGGCGTTTATTAGACC-3′ and reverse: 5′-TCAATTCCTTTAAGTTTCAGCC-3′) was used to amplify the *18S rRNA* gene (1093 bp) in a conventional PCR for the detection of *Theileria* spp. [29]. The PCR was carried out in a Thermal Cycler (Bio-Rad Laboratories Inc., Hercules, CA, USA) containing a reaction mixture of 25 μL comprised of 13 μL Dream *Taq* PCR MasterMix (2×) (Thermo Fisher Scientific, Inc., Waltham, MA, USA); 2 μL of primers (1 μL each forward and reverse); 2 μL (50 ng/μL) of extracted genomic DNA; and 8 μL “nuclease-free” water. The conditions for thermal cycling in the PCR were: initial denaturation at 94 °C for 3 min followed by 40 cycles of 94 °C for 30 s, 58 °C for 60 s and 72 °C for 60 s, and final extension at 72 °C for 5 min [29]. Each PCR experiment contained a negative control (PCR water instead of template DNA) and positive control (*T. annulata* DNA) [19]. The amplified PCR products were run on a 1.5% agarose gel, dyed with 2 μL of ethidium bromide, and observed by a gel documentation system (BioDoc-It^TM^ Imaging Systems UVP, LLC, Upland, CA, USA) (Figure S1).

### 2.5. DNA Sequencing and Phylogenetic Analysis

The GeneClean II kit (Qbiogene, Carlsbad, CA, USA) was used to purify the PCR products following the manufacturer’s protocol. A total of 36 (two from each breed in each district) amplified PCR products were submitted for bidirectional sequencing (Macrogen, Inc., Seoul, Korea). The obtained sequences were subjected to SeqMan V. 5 (DNASTAR, Inc., Madison, WI, USA) for trimming to remove the contaminated nucleotide and primer regions. The trimmed sequences were submitted to BLAST (Basic Local Alignment Search Tool) [30] at NCBI (National Center for Biotechnology Information). Homologous sequences were downloaded from NCBI and aligned with the obtained sequences and an outgroup in BioEdit V. 7.0.5 (Raleigh, NC, USA) [31]. The phylogenetic tree was constructed by using the maximum-likelihood model (1000 bootstrap replicons) in MEGA-X (Molecular Evolutionary Genetics Analysis) [32].

### 2.6. Statistical Analyses 

Statistical analyses were performed in the GraphPad Prism V. 5 (Inc., San Diego, CA, USA) [33]. The variables were classified into two or more categories to determine a significant difference and relative risk (RR) [34] between the occurrence of *Theileria* infection and the potential risk factors using a chi-square test and logistic regression analysis, respectively. The analysis was considered to be significant at a 95% confidence interval (CI) [35] and *p*-value < 0.05.

## 3. Results

### 3.1. Collected Blood Samples

A total of 555 blood samples were collected from six districts, including Peshawar (80, 14.4%); Charsadda (112, 20.2%); Malakand (88, 15.8%); Lower Dir (90, 16.2%); Upper Dir (75, 13.5%); and Bajaur (110, 19.8%) of KP, Pakistan.

### 3.2. Microscopic Examination

The microscopic examination of the collected blood samples showed *Theileria* infection in the cattle hosts of the selected regions. The presence of a sting-ring, oval, rod, or comma-shaped parasite was considered as positive for *Theileria* spp. Among the collected 555 blood samples, 136 (24.5%) were microscopically found to be positive for *Theileria* spp. The highest infection was observed in blood samples collected from Lower Dir (28/90, 31.1%), followed by Charsadda (34/112, 30.35%); Upper Dir (28/75, 29.3%); Malakand (21/88 23.8%); Peshawar (15/80, 18.75%); and Bajaur (16/110, 14.5%) (Table 1).

### 3.3. Molecular Confirmation of Theileria Infection 

Among the collected blood samples, 294 (53%) (including 136 microscopically positive) were confirmed to be positive for *Theileria* species by using a conventional PCR to amplify *18S rDNA* sequences, using species-specific primers to identify the prevalent *Theileria* spp. from all selected districts.

### 3.4. Sequence and Phylogenetic Analyses

**Figure 2:**
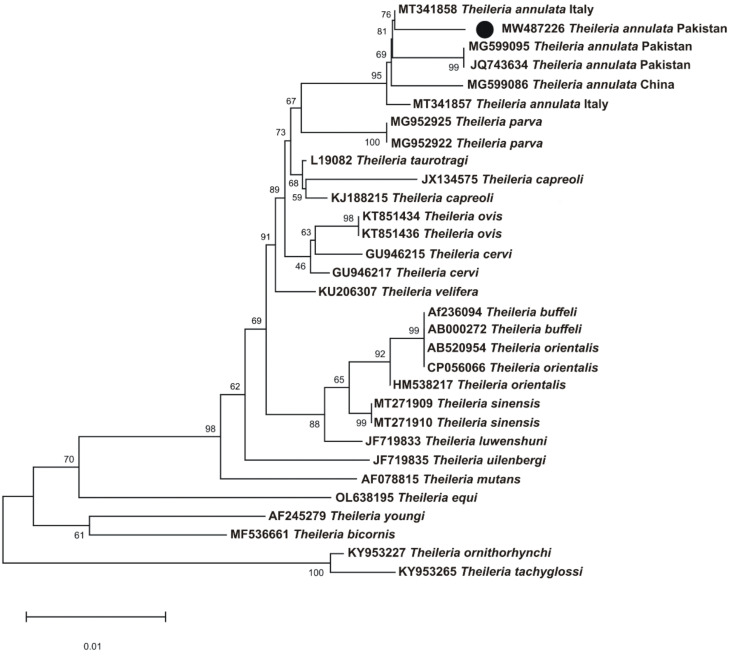
The maximum likelihood model at 1000 bootstrap replicons was used to construct a phylogenetic tree for the *18S rDNA* sequences of *T.* *annulata*. *Theileria ornithorhynchid*, and *Theileria tachyglossi 18S rDNA* sequences were utilized as an outgroup. A black circle has been used to label the obtained sequences in this study (MW487226).

The obtained sequences with 100% identity were considered as a single sequence. The BLAST results of the obtained *18S rDNA* sequences of 1000 bp revealed a 99.5% maximum identity with *T. annulata*. A total of 31 sequences of the *18S rDNA* sequences for *T. annulata* were downloaded from GenBank in FASTA format based on maximum identity with query sequences. The *18S rDNA* sequence for *T. annulata* was clustered in a phylogenetic tree with the sequences reported from Pakistan, China, and Italy (Figure 2). The *18S rDNA* sequence of *T. annulata* was deposited to GenBank (MW487226).

### 3.5. Risk Factors Associated with Theileria Infection

Collected data about host (age, gender, breeds); management practices (feeding system, hygienic measures, farming system, and stall system); and seasonal patterns between summer (June, July, and August) and winter (December, January, and February) were analyzed, which assisted in identifying various risk factors associated with *Theileria* infection. Adult cattle of 2 to 6 years of age were found to be more highly infected (216/360, 60%) than young cattle of less than 2 years of age (78/195, 40%). The gendered prevalence was a significant risk factor in target sites, as female hosts were at more risk (218/377, 57.8%) than males (76/178, 42.6%). Breed-wise, the highest infection rate was observed in Holstein Friesian (120/180, 66.6%), followed by Jersey (106/176, 60.2%) and Sahiwal cattle (68/199, 34.2%). In the current study, different management practices such as the feeding system were observed as a significant risk factor, in which free-grazing cattle were found to be more highly infected (190/412, 42.2%) than stall feeding and tied cattle (104/143, 72.7%). Cattle in areas with effective hygienic measures were less affected by the infection (48/150, 32%) than those kept in unhygienic conditions (246/405, 60.7%). The cattle in the combined farming system were more highly infected (165/255, 64.5%) than in the isolated farming system (129/300, 43%). The infection rate was found to be higher in the summer (215/350, 61.4%) as compared to the winter (79/205, 38.5%) season (Table 2).

## 4. Discussion

Studies have shown the negative impact of *T. annulata* infection on livestock in some regions of Pakistan [36]. The majority of these studies were based on a microscopic-diagnosis of the infection, lacking sufficient molecular information about the genetic diversity of prevalent *Theileria* spp. [1,36]. The current study provides a detailed insight into the epidemiology, genetic diversity, and risk factors associated with these etiological agents for bovine theileriosis in KP, Pakistan. Blood samples collected from symptomatic cattle were investigated by microscopic examination and PCR for amplification of the *18S rDNA* sequence that revealed a high prevalence of *T. annulata* in cattle. Prevalence of *Theileria* infection was significantly associated with various factors such as age, gender, breeds, feeding system, hygienic measures, farming system, stall system, and different seasons of the year.

Microscopic examination is a rapid and low-cost method for detecting and diagnosing several infectious agents, including *Theileria* spp. [37]. However, this method has often been less sensitive in diagnosing and accurately identifying various *Theileria* species [1,20]. During this study, more than half of the blood samples that detected negative for *Theileria* species through microscopic examination were found to be positive by a PCR test and sequencing. These results indicate that using the *18S rDNA* sequence in a PCR test to detect *Theileria* spp. in blood samples has a higher accuracy than microscopy [38]. Based on the results, it is suggested that microscopic examination in combination with PCR and DNA sequencing should be adopted for the detection and accurate identification of *Theileria* spp.

In a phylogenetic tree, the *18S rDNA* sequence of *T. annulata* clustered with the sequences from Pakistan, China, and Italy. The species-specific primers used to characterize the *18S rDNA* sequences for *T. annulata* were able to identify this parasite accurately. The *18S rDNA* sequences of *T. annulata* hold significance in describing genetic diversity due to the presence of hypervariable regions, which are crucial for determining evolutionary patterns [18].

Adult cattle (2 to 6 years old) were more highly infected than young cattle (≤2 years old). The high level of infection in adult cattle may be due to their regular exposure to questing ticks during free-grazing, and weak immune responses [39,40,41]. Early colostrum intake in calves has been suggested to enhance immunity against various pathogens, including *Theileria* spp. [42,43]. Gender-wise, female cattle were more highly infected than male cattle. The suppression of immunity during pregnancy and lactation in female cattle may be the possible reason for this difference in prevalence [44,45]. Breed-wise, the highest level of infection was observed in the Holstein Friesian, followed by Jersey and Sahiwal cattle. These findings suggest that importing exotic cattle breeds (Holstein Friesian) into Pakistan has enhanced the prevalence of *Theileria* infection [36,46].

Furthermore, the exposure of indigenous breeds to local ticks and tick-borne pathogens is common and might assist in developing protective immunity to fight against these infectious agents [14,47]. A higher level of infection was detected in free-grazing cattle than tied cattle. The contact of healthy cattle with infected cattle during free-grazing has been shown as a critical risk factor in enhancing the burden and spread of *Theileria* infection [13,39]. Congested stalls and unhygienic conditions were found to be potential risk factors responsible for high *Theileria* infection. This increased rate of infection may be due to the close contact of infected cattle with healthy cattle and the traumatic conditions cattle face in crowded stalls that suppress immunity to various infections [13,39,48]. A high prevalence of *Theileria* infection was recorded in the summer compared to the winter season. Warm and humid conditions favor ticks’ reproduction, growth, and dispersal, which may assist their quest and access to cattle hosts [3,49]. This region has recorded a high prevalence of various tick species, including vectors for *Theileria* species, during summer.

## 5. Conclusions

This study provides information about the prevalence and risk factors associated with theileriosis in different regions of Pakistan. In addition, some information was provided about the molecular characterization of *Theileria* spp. infecting cattle in selected districts of KP, Pakistan. PCR and sequencing are essential approaches for detecting and identifying *Theileria* spp. *T.* *annulata* was the dominant species detected in the investigated areas. Risk factors such as host age, gender, breeds, feeding system, hygienic measures, farming system, stall system, and different seasonal patterns were potential determinants that significantly enhanced the chances of *Theileria* infection. Using conventional microscopic examination in combination with molecular approaches will assist in adopting essential measures for the early detection and control of *Theileria* infection, in order to enhance livestock production.

## Figures and Tables

**Table 1:** Comparison of microscopic examination and PCR for detecting *Theileria annulata* in collected blood samples.

Districts	Cattle Breeds	Blood Sample (%)	Microscopy +ve (%)	95% CI	*p* Value	PCR +ve (%)	95% CI	*p* Value
**Peshawar**	Holstein Friesian	21 (26.2)	6 (7.5)	−39.7–6.4	0.0449	11 (13.75)	−2–2	0.05
	Jersey	24 (30)	5 (6.25)			12 (15)		
	Sahiwal	35 (43.7)	4 (5)			17 (21.25)		
	Total	80 (100)	15 (18.75)			40 (50)		
**Charsadda**	Holstein Friesian	38 (33.9)	12 (10.7)	−29–6.30	0.0260	18 (16.07)	−2–17	0.31
	Jersey	41 (36.6)	16 (14.2)			15 (13.39)		
	Sahiwal	33 (29.4)	6 (5.3)			19 (16.96)		
	Total	112 (100)	34 (30.35)			52 (46.4)		
**Malakand**	Holstein Friesian	26 (29.5)	8 (9.09)	−27.8–(−2.89)	0.0170	21 (23.8)	−2–3	0.24
	Jersey	30 (34.1)	7 (7.9)			13 (14.7)		
	Sahiwal	32 (36.3)	6 (6.8)			17 (19.3)		
	Total	88 (100)	21 (23.8)			51 (57.9)		
**Lower Dir**	Holstein Friesian	40 (44.4)	12 (13.3)	−27–4.6	0.046	23 (25.5)	−3–15	0.06
	Jersey	26 (28.8)	6 (6.6)			18 (20)		
	Sahiwal	24 (26.6)	10 (11.1)			13 (14.4)		
	Total	90 (100)	28 (31.1)			54 (60)		
**Upper Dir**	Holstein Friesian	15 (20)	5 (6.6)	−24–3	0.041	12 (16)	−2–3	0.25
	Jersey	28 (37.3)	9 (12)			22 (29.3)		
	Sahiwal	32 (42.6)	8 (10.6)			12 (16)		
	Total	75 (100)	22 (29.3)			46 (61.3)		
**Bajaur**	Holstein Friesian	48 (43.6)	9 (8.1)	−37–(−14)	0.005	27 (24.5)	−2–18	0.31
	Jersey	27 (24.5)	3 (2.7)			12 (10.9)		
	Sahiwal	35 (31.8)	4 (3.6)			12 (10.9)		
	Total	110 (100)	16 (14.5)			51 (46.36)		
**Overall Total**		**555 (100)**	**136 (24.5)**			**294 (53)**		

**Table 2:** Assessment of various risk factors associated with *Theileria* infection.

Variable		Total Examined	Positive Sample (%)	95% CI	RR	*p* Value
**Age**	≤2 years	195	78/195 (40)	1.26–1.77	1.5	<0.0001
	>2 years–6 years	360	216/360 (60)	–	–	
**Gender**	Female	377	218/377 (57.8)	–	–	0.0006
	Male	178	76/178 (42.6)	0.61–0.86	0.727	
**Breeds**	Holstein Friesian	180	120/180 (66.6)	–	–	0.0001
	Jersey	176	106/176 (60.2)	0.63–1.09	0.833	
	Sahiwal	199	68/199 (34.2)	0.40–0.64	0.509	
**Feeding system**	Free-grazing	412	190/412 (42.2)	–	–	<0.0001
	Stall fed	143	104/143 (72.7)	1.4 9–2.62	1.976	
**Hygienic measures**	Hygienic	150	48/150 (32)	–	–	<0.0001
	Unhygienic	405	246/405 (60.7)	1.48–2.06	1.754	
**Farming system**	Combine	255	165/255 (64.8)	–	–	<0.0001
	Isolated	300	129/300 (43)	1.284–1.764	1.505	
**Stall system**	Congested	218	150/218 (68.8)	–	–	<0.001
	Open	337	144/337 (42.7)	1.3–1.8	1.6	
**Seasons**	Summer	350	215/350 (61.4)	–	–	<0.0001
	Winter	205	79/205 (38.5)	0.52–0.74	0.628	
**Total samples**		**555**	**294/555 (53)**			

## Data Availability

Details regarding data supporting reported results can be found https://www.ncbi.nlm.nih.gov/nuccore/?term= (accessed on 26 June 2022).

## Supplementary Materials

The following supporting information can be downloaded at: https://www.mdpi.com/article/10.3390/microorganisms10081614/s1, File S1: Sample collection Performa; Figure S1: The PCR based amplified products containing fragment (1093 bp) DNA amplified through species-specific primers (Lane 1-6) for *Theileria annulata*. The positive control (*Theileria annulata*) is shown as PC and negative control as NC (PCR water instead of template DNA).
