# Supplementary material for: Molecular Characterization and Assessment of Risk Factors Associated with Theileria annulata Infection
Source: Microorganisms. 2022 Aug 9;10(8):1614. doi: 10.3390/microorganisms10081614 (PMC9412660; doi:10.3390/microorganisms10081614)
Supplement: Supplementary file 1 [file microorganisms-10-01614-s001.zip › Figure S1.pdf]

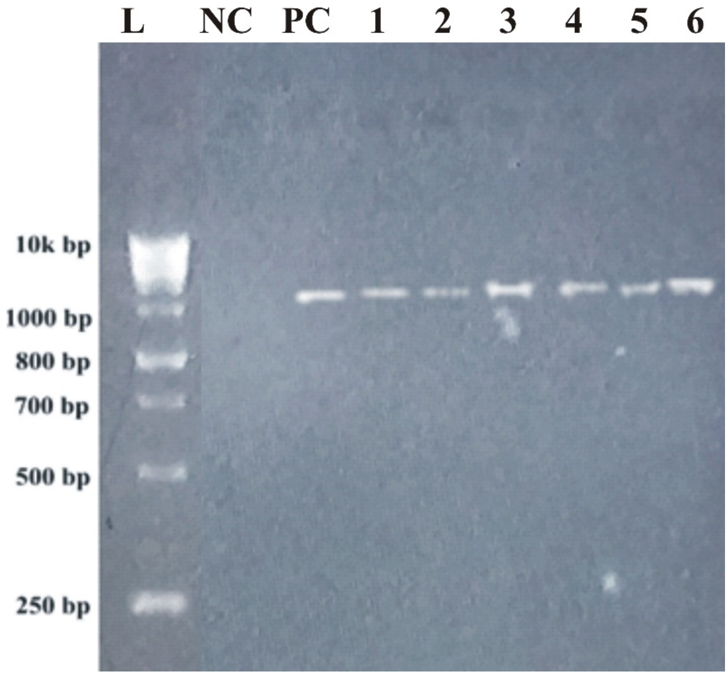

Figure S1. The PCR based amplified products containing fragment (1093 bp) DNA amplified through species-specific primers (Lane 1-6), for *Theileria annulata*, respectively. The positive control (*Theileria annulata*) is shown as PC and negative control as NC (PCR water instead of template DNA).
