# Supplementary material for: Molecular Characterization and Assessment of Risk Factors Associated with Theileria annulata Infection
Source: Microorganisms. 2022 Aug 9;10(8):1614. doi: 10.3390/microorganisms10081614 (PMC9412660; doi:10.3390/microorganisms10081614)
Supplement: Supplementary file 1 [file microorganisms-10-01614-s001.zip › File S1.pdf]

Sample collection Performa

Questionnaire was divided into two parts which were as follows: (a) host analysis (b) Risk factors analysis.

Part A. Information related to cattle Host and management practices

1- Signs and symptoms

a) Symptomatic host

b) Asymptomatic host

2-Infection history

a) Frist time infected

b) Several time infected

3-Ticks infestation

a) Present
b) Absent

Part B. Information related to associated Risk factors

1-Different characteristics of host

a) Indigenous

b) Exotic

c) Age group < 2 year and > 2 years- 6 year

d) Gender- Male, female

e) Breed-

Holstein Frisian,

Jersey

Sahiwal

2-Management practices

a) Feeding system

Free grazing

Stall fed

b) Hygienic measures

Hygienic

Unhygienic

c) Farming system

Combine

Separate

d) Stall pattern

Congested

Open

3 Season of the year and dates

Winter

Summer
